# Patents and regulatory exclusivities on FDA-approved insulin products: A longitudinal database study, 1986–2019

**DOI:** 10.1371/journal.pmed.1004309

**Published:** 2023-11-16

**Authors:** Anders Olsen, Reed F. Beall, Ryan P. Knox, Sean S. Tu, Aaron S. Kesselheim, William B. Feldman

**Affiliations:** 1 Program On Regulation, Therapeutics, And Law, Division of Pharmacoepidemiology and Pharmacoeconomics, Department of Medicine, Brigham and Women’s Hospital and Harvard Medical School, Boston, Massachusetts, United States of America; 2 Department of Medicine, Beth Israel Deaconess Medical Center and Harvard Medical School, Boston, Massachusetts, United States of America; 3 Department of Community Health Sciences, Cumming School of Medicine, University of Calgary, Calgary, Canada; 4 Harvard-MIT Center for Regulatory Science, Boston, Massachusetts, United States of America; 5 West Virginia University College of Law, Morgantown, West Virginia, United States of America; 6 Division of Pulmonary and Critical Care Medicine, Department of Medicine, Brigham and Women’s Hospital and Harvard Medical School, Boston, Massachusetts, United States of America

## Abstract

**Background:**

Insulin is the primary treatment for type 1 and some type 2 diabetes but remains costly in the United States, even though it was discovered more than a century ago. High prices can lead to nonadherence and are often sustained by patents and regulatory exclusivities that limit competition on brand-name products. We sought to examine how manufacturers have used patents and regulatory exclusivities on insulin products approved from 1986 to 2019 to extend periods of market exclusivity.

**Methods and findings:**

We used the publicly available Food and Drug Administration (FDA) Approved Drug Products with Therapeutic Equivalence Evaluations (Orange Book) to identify all approved biosynthetic insulin products. Individual products approved under the same New Drug Application (NDA)—e.g., a vial and pen—were considered as separate products for the purposes of analysis. We recorded all patents and regulatory exclusivities listed in the Orange Book on each product and used Google Patents to extract the timing of patent application and whether patents were obtained on delivery devices or others aspects of the product. The primary outcome was the duration of expected protection, which was determined by subtracting the FDA approval date for each product from its last-to-expire patent or regulatory exclusivity (whichever occurred later). We performed a secondary analysis that considered overall protection on insulin lines—defined as groups of products approved under the same NDA with the same active ingredients manufactured by the same company. We also examined competition from follow-on insulin products—defined as products approved with the same active ingredients as originators but manufactured by different companies (approved via a specific drug approval pathway under section 505(b)(2) of the Food, Drug, and Cosmetic Act). During the study period, the FDA approved 56 individual products across 25 different insulin lines and 5 follow-ons across 3 different insulin lines. Thirty-three (59%) of the 56 products were drug-device combinations. Manufacturers of 9 products approved during the study period obtained patents filed after FDA approval that extended their duration of expected protection (by a median of 6 years). Approximately 63% of all patents on drug-device combinations approved during the study period were related to delivery devices. The median duration of expected protection on insulin products was 16.0 years, and the median protection on insulin lines was 17.6 years. An important limitation of our analysis is that manufacturers may continue to add patents on existing insulin products while competitors may challenge patents; therefore, periods of protection may change over time.

**Conclusions:**

Among several strategies that insulin manufacturers have employed to extend periods of market exclusivity on brand-name insulin products are filing patents after FDA approval and obtaining a large number of patents on delivery devices. Policy reforms are needed to promote timely competition in the pharmaceutical market and ensure that patients have access to low-cost drugs.

## Abstract 2

Anders Olsen and colleagues explore patenting on insulin products by the Federal Drug Agency (FDA) and the impact on market exclusivity and drug prices.

## Author summary

**Why was this study done?:**

- Patents and regulatory exclusivities on brand-name drugs can support high prices by limiting competition.
- We sought to describe how manufacturers have used patents and regulatory exclusivities on insulin products approved in the United States from 1986 to 2019 to establish periods of market exclusivity.

**What did the researchers find?:**

- The Food and Drug Administration (FDA) approved 56 brand-name originator insulin products during the study period. The median duration of expected patent protection on these products was 16.0 years.
- Insulin manufacturers have extended periods of market exclusivity by filing many patents on their products, including many after FDA approval.
- Of all patents on the 33 drug-device combinations in the cohort, 63% were related to delivery devices.

**What do these findings mean?:**

- Policy reforms are needed to promote competition in the pharmaceutical market and ensure that patients have timely access to drugs at fair prices.
- A limitation of this work is that realized periods of market exclusivity may shift in the future, because brand-name firms may add more patents over time and competitors may challenge existing patents.

## Introduction

Insulin remains a central treatment modality in the management of diabetes. Patients with type 1 diabetes typically require lifelong insulin replacement therapy [1,2], while those with type 2 diabetes who cannot achieve adequate glycemic control with lifestyle modification and non-insulin therapies (e.g., metformin, glucagon-like peptide 1 [GLP-1] receptor agonists, sodium-glucose cotransporter 2 [SGLT2] inhibitors) require insulin supplementation as their disease progresses [3]. Of the more than 500 million people worldwide with diabetes [4], an estimated 71 million rely on insulin [5]. In the United States, where the prevalence of diabetes now exceeds 10% [6], more than 7 million people require insulin therapy [7]. Yet, while insulin was discovered more than a century ago, prices for treatment with insulin remain high [8]. Roughly 1 in 6 patients in the US ration their insulin due to cost, leading to avoidable hospitalizations and life-threatening complications [9]. With high prices for other diabetes therapies, particularly GLP-1 receptor agonists and SGLT2 inhibitors [10], diabetes remains the most expensive chronic disease in the US [11].

The insulin market is dominated by 3 manufacturers—Eli Lilly, Sanofi, and Novo Nordisk—that represent over 90% of all global sales and were, until recently, the sole suppliers of insulin to the US [12]. A 2021 Congressional report found that, for decades, these 3 major manufacturers raised US list prices on insulin year over year, often in tandem with one another [7]. Some policymakers have pointed to the patent and regulatory system as one factor that has limited competition and allowed manufacturers to raise prices [13].

Patents are government-granted monopolies that last 20 years and are issued by the US Patent and Trademark Office (USPTO) for novel inventions that are not obvious over existing technology. Manufacturers list key patents on pharmaceutical products in the Food and Drug Administration’s (FDA) *Approved Drug Products with Therapeutics Equivalence Evaluations* (Orange Book), and the FDA cannot approve generic versions of drugs for marketing until patents have expired or have been successfully challenged. The FDA serves in a purely “ministerial” role relating to patents that manufacturers submit for inclusion in the Orange Book—recording and publishing updated lists of submitted patents, but not assessing their validity [14]. Brand-name firms often list a large number of patents on their products, which can increase the scope of patent challenges, and these firms may sue competitors when patent challenges are filed, which can be costly and lead to delays in generic drug approval.

There is growing concern that the USPTO’s standards for granting new patents have been too low [15,16]. In the pharmaceutical market, manufacturers often seek patents not only on the underlying active ingredients in the drugs but also on peripheral aspects such as the excipients and methods of use. Apart from patents, drug manufacturers are also protected from competition by regulatory exclusivities, which are granted by the FDA and vary in length depending on the exclusivity that is granted (e.g., exclusivities granted for approval of new chemical entities last 5 years, while exclusivities granted for older drugs with new dosage formulations last 3 years) [14]. Together, patents and regulatory exclusivities can serve to block generic competition and sustain high drug prices in the US pharmaceutical market.

Drug-device combinations, like many insulin products, are particularly susceptible to extensive patenting since manufacturers can list patents on the devices that deliver medications [17,18]. For example, a recent study of inhalers, another important class of drug-device combinations, found that more than half of patents were listed on the delivery device and that manufacturers frequently launched products in new devices with the same active ingredients as existing products (so-called “device hopping”) [19]. A federal appeals court recently found that certain types of device patents listed in the Orange Book—namely, those with no mention of active ingredients in their claims—may be improperly listed [20], and yet more than three-quarters of device patents on inhalers approved from 1986 to 2020 made no such mention of active ingredients [21]. While new devices can provide benefits to patients, such as simplifying methods for self-administration and increasing adherence, patents on these devices can lead to delayed competition.

The patent system is designed to reward innovation by protecting patent holders from competition for a set period of time. Identifying and addressing practices that serve to prolong such protection is critical for ensuring that the right balance is struck between genuine innovation and price-lowering competition. We therefore examined the evolution of patents and regulatory exclusivities on insulin products since the first modern, biosynthetic versions were released in the 1980s. To understand the impact of these protections, we also analyzed the timing of “follow-on” competitor insulin products. Regulations precluded the FDA from approving generic versions of insulin during the study period; follow-on insulins were instead approved via a specific drug approval pathway under section 505(b)(2) of the Food, Drug, and Cosmetic Act [22]. This pathway streamlines the approval process for products with already-marketed active ingredients and allows follow-on manufacturers to rely in part on clinical studies performed by the originator manufacturer. As with generic products, manufacturers seeking 505(b)(2) approval must challenge any active patents listed in the Orange Book at the time of application [23,24].

The current study builds on earlier work identifying a high number of device patents on insulin products [25,26], but these earlier studies only included patents from 3 snapshots in time (2004, 2014, and 2020) and did not analyze how manufacturers have added patents to their products after FDA approval, engaged in device hopping, relied on device patents disconnected from active ingredients, or, more generally, used these strategies to limit competition from follow-on products. A deeper understanding of these industry dynamics is crucial not only for regulators and lawmakers to design policies that prevent manufacturers from delaying competition on insulin in the future but also for advancing our knowledge of patenting practices on drug-device combinations. The USPTO and FDA have identified a set of strategies to enhance collaboration to limit patent abuses [27], and drug-device combinations have become a particular focus for lawmakers [28]. We identified all patents and regulatory exclusivities on insulin products approved by the FDA between 1986 and 2019 and described how these patents and exclusivities extended periods of market exclusivity.

## Methods

### Cohort identification

We used the publicly available FDA Orange Book to identify insulin products approved from 1986 to 2019. We selected 1986 as the start of the study period because this is when consecutive Orange Books listing patent information first became available [14]. Every insulin product listed in the Orange Book is associated with a New Drug Application (NDA). We considered individual products sold under the same NDA as separate products for the purposes of analysis. For example, Lantus (insulin glargine) was approved as a vial in 2000 and as a pen in 2007 under the same NDA. Because the device was a major barrier to competition for Lantus, we considered the vial and pen as separate products. Further, this helped us differentiate the more novel patent practices on drug-device combination products compared to drug products. We refer to products under the same NDA—which have the same active ingredients and are manufactured by the same company—as a single insulin line (S1 Methods).

We excluded animal-derived insulin products (S1 Table), as our focus was on modern biosynthetic insulins used today (animal-derived insulins, which were associated with immune-mediated reactions, have all been removed from the US market) [29]. The 3 earliest biosynthetic insulins, Humulin R, Humulin L, and Humulin N, were excluded from the cohort since these were approved prior to 1986 (S2 Table). We ended the study period with drugs approved in 2019, because the FDA began regulating all insulins as biologics (rather than small-molecule drugs) in March 2020, which meant that these products and their patents were removed from subsequent editions of the Orange Book [30]. Insulin products in our cohort were categorized based on their onset of action: rapid-acting, short-acting, intermediate-acting, long-acting, and mixtures (including combined long-acting insulin-incretin mimetics, combined intermediate-rapid-acting insulins, and combined intermediate-short-acting insulins) (S1 Methods).

### Data extraction

We used a database from the National Bureau for Economic Research (NBER) containing Orange Book listings to identify patents and regulatory exclusivities and their dates of expiration for all insulin products [31,32]. This NBER resource has been used in other analyses of the US patent system [33,34]. We then manually confirmed that each NBER patent and exclusivity listing on insulin products matched the corresponding Orange Book listing [35]. For years with no NBER data (2017 to 2020), we relied solely on manual extraction of data from the Orange Book. For each regulatory exclusivity listed in the Orange Book, we recorded the category of exclusivity and its duration. We performed our search for patents and regulatory exclusivities in every annual edition of the Orange Book through the 2020 edition (when drugs approved in 2019 would first appear).

We used Google Patents to obtain detailed information on each patent, including the patent title, claims (which define the scope of what the patent covers), application date, and priority date (which specifies the start of 20-year patent terms and is tied to the date when the first member in the patent family is filed). We determined whether each patent was associated with an insulin delivery device (“device patent”) or another aspect of the product, such as the active ingredient or an excipient (“non-device patent”). We also determined whether the application for each patent had been filed with the USPTO before FDA approval of a given product (“pre-approval patent”) or after approval (“post-approval patent”) and whether each regulatory exclusivity was granted at the time of FDA approval (“approval exclusivity”) or after FDA approval (“post-approval exclusivity”). For all device patents listed on insulin products, we assessed whether any of its claims made mention of insulin (the active ingredient) or its molecular structure. Two reviewers independently characterized each patent.

### Duration of protection from competition

To determine the duration of expected protection for each insulin product, we subtracted the FDA approval date from the last-to-expire patent or regulatory exclusivity listed in the Orange Book. Individual insulin products were the primary focus of our analysis. Thus, if both a vial and pen were listed under the same NDA, we analyzed the expected protection for the vial separately from expected protection for the pen. We also performed an analysis in which we considered the overall duration of expected protection for products that were covered under the same NDA (i.e., the same insulin line).

To understand the specific role of device patents in extending market exclusivity on drug-device combinations, we calculated the added protection from device patents by subtracting the last-to-expire non-device patent from the last-to-expire device patent. Given specific concerns recently raised by courts about device patents that make no mention of active ingredients, we also separately quantified the protection afforded by these types of device patents (with no mention of insulin in the claims) over and above the protection afforded by other patents (device patents referencing active ingredients or non-device patents, whichever expired later).

### Sensitivity analysis

We performed a sensitivity analysis in which we considered early competition from follow-on competitors (products with the same active ingredient made by different manufacturers) approved via the 505(b)(2) pathway. In this sensitivity analysis, we calculated the duration of patent protection on originators from the time of FDA approval to the last-to-expire patent or regulatory exclusivity or approval of the first follow-on (whichever occurred first).

### Statistical analyses

All analyses were performed using R Core Team 3.6.3 and Excel 16.59 (Microsoft). We performed descriptive analyses to quantify the number of patents and regulatory exclusivities per product and used data visualization methods to explore trends over time. We did not perform formal hypothesis testing on the primary outcome (expected duration of protection from patents and regulatory exclusivities), as the goal was to describe periods of protection for products across the cohort. Institutional Review Board approval was not required since the study did not involve human participants in its analysis or data collection.

## Results

The FDA approved 56 products across 25 originator brand-name insulin lines (Tables 1 and S3) and 5 follow-ons across 3 different insulin lines (Tables 2 and S4) from 1986 to 2019. Of the 25 originator insulin lines, 12 (48%) were manufactured by Novo Nordisk, 7 (28%) by Eli Lilly, 4 (16%) by Sanofi, 1 (4%) by Pfizer, and 1 (4%) by MannKind. Thirty-three (59%) of the 56 insulin products were drug-device combinations, while the other 23 (41%) were drug products (e.g., vials). More than three-quarters of the drug-device combinations (26/33) were one-time use products, while the remainder were reusable (with refillable cartridges) [36].

**Table 1:** Biosynthetic originator insulins approved from 1986–2019.

Brand	Insulin	First approval	Number of products	Number of drug-device combinations
**Rapid-acting insulins**				
Humalog	lispro	6/14/1996	4^a^	3
Velosulin BR	human	07/19/1999	1	0
Novolog	aspart	06/07/2000	5	3
Apidra	glulisine	04/16/2004	3	1
Exubera	human	01/27/2006	2	2
Afrezza	human	06/27/2014	3	3
Fiasp	aspart	09/29/2017	3	1
**Short-acting insulins**				
Humulin BR	regular	04/28/1986	1	0
Humulin U	regular	06/10/1987	2	0
Novolin L	regular	06/25/1991	1	0
Novolin R	regular	06/25/1991	1	1
**Intermediate-acting insulin**				
Novolin N	NPH	07/01/1991	1	1
**Long-acting insulins**				
Lantus	glargine	04/20/2000	2	1
Levemir	detemir	06/16/2005	5	4
Toujeo	glargine	02/25/2015	2	2
Tresiba	degludec	09/25/2015	3	1
**Long-acting and incretin mimetic**				
Xultophy 100/3.6	glargine/lixisenatide	11/21/2016	1	1
Soliquo 100/33	glargine/lixisenatide	11/21/2016	1	1
**Intermediate-acting and rapid-acting insulin mix**				
Humalog 50/50	lispro protamine/lispro	12/22/1999	3	2
Humalog 75/25	lispro protamine/lispro	12/22/1999	3	2
Novolog 70/30	aspart protamine/aspart	11/01/2001	4	1
Ryzodeg 70/30	degludec/aspart	09/25/2015	1	1
**Intermediate and short-acting insulins**				
Humulin 70/30	NPH/regular	04/25/1989	2	1
Novolin 70/30	NPH/regular	06/25/1991	1	1
Humulin 50/50	NPH/regular	04/29/1992	1	0

^a^Humalog insulin includes 3 products listed under one New Drug Application and 1 product under another (see S1 Methods).

**Table 2:** Biosynthetic follow-on insulins approved from 1986–2019.

Brand	Insulin	First approval	Number of products	Number of drug-device combinations
**Rapid-acting insulins**				
Admelog	lispro	12/11/2017	3	1
Myxredlin	human	06/20/2019	1	0
**Long-acting insulins**				
Basaglar	glargine	12/16/2015	1	1

The 3 follow-on lines approved via 505(b)(2) applications were Basaglar (glargine, Eli Lilly) in 2015; Admelog (lispro, Sanofi) in 2017; and Myxredlin (human, Baxter) in 2019. Basaglar was released only as a drug-device combination (KwikPen), Admelog as a drug-device combination (SoloStar) and vial (in 2 different sizes), and Myxredlin as an intravenous infusion bag for inpatient use.

### Patents and exclusivities on originator insulins at the time of approval

The 56 individual originator products approved across the 25 brand-name insulin lines received 28 regulatory exclusivities at the time of FDA approval (see S5 Table for a description of these exclusivities). Twenty-four products received 1 exclusivity, and 2 products received 2 exclusivities. These 28 regulatory exclusivities included 11 (39%) for new chemical entities, 10 (36%) for new products, 6 (21%) for new combinations, and 1 (4%) for new patient populations.

**Fig 1:**
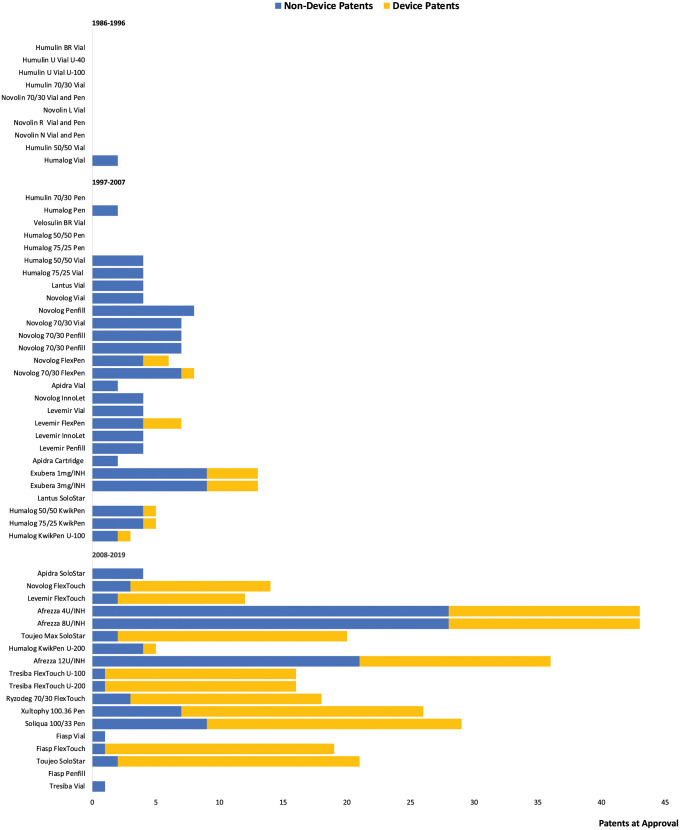
Device and non-device patents listed on brand-name originator insulins at the time of approval. This figure shows the number of non-device patents (blue bars) and device patents (yellow bars) that manufacturers filed on their products before Food and Drug Administration (FDA) approval. Products in the figure with no bars had no patents listed in the FDA Orange Book that were filed before FDA approval.

Across the 56 individual originator insulins, manufacturers listed 454 patents filed with the USPTO before FDA approval. The median number of pre-approval patents listed per product increased from 0 (IQR 0 to 0) during the first third of the study period (1986 to 1996) to 4 (IQR 2 to 7) during the second third (1997 to 2007) to 17 (IQR 7 to 25) during the final third (2008 to 2019) (Fig 1; see S6 Table for similar findings when analyzing 5-year increments). Overall, manufacturers listed a median of 5 (IQR 2 to 13) pre-approval patents per product. Of the total 454 pre-approval patents, 224 (49%) were device patents and the remainder were non-device patents. The median number of device patents listed per product increased from 0 (IQR 0 to 0) in 1986 to 1996 and 0 (IQR 0 to 1) in 1997 to 2007 to 15 (IQR 3 to 17) in 2008 to 2019.

The median expected market exclusivity based on the listed patents and regulatory exclusivities at FDA approval was 15.6 years (IQR 10.3 to 17.4).

### Patents and exclusivities on originator insulins after FDA approval

Manufacturers for the 56 individual originator brand-name products in the cohort obtained 10 pediatric exclusivities (S7 Table) after approval and 27 other regulatory exclusivities—8 (30%) for new patient populations, 3 (19%) for new routes of administration, 1 (4%) for new products, 2 (7%) for new combinations, and the remaining 13 (48%) for unique indications and dosing changes (S8 Table). The median number of exclusivities obtained after approval per product was 1 (IQR 0 to 2).

Manufacturers listed a total of 107 patents in the Orange Book after approval. Eighty-four (79%) were device patents. The Apidra SoloStar and Lantus SoloStar had the most post-approval patents (18 each), all of which were on the delivery devices. Eighty-seven (81%) post-approval patents had priority dates before FDA approval, while 20 (19%) had priority dates after FDA approval. The median number of post-approval patents per product was 0 (IQR 0 to 3).

Nine products received extended protection from their post-approval patents. The median duration of extension on these 9 products was 6.0 years (IQR 3.0 to 11.3).

### Overall duration of protection on originator insulins

When considering patents and regulatory exclusivities both at the time of FDA approval and added later, the median duration of expected protection for products in the cohort was 16.0 years (IQR 10.3 to 18.1) from FDA approval. The long-acting insulin/incretin mimetic and rapid acting classes had the longest durations of protection by insulin class at 17.1 years each (S9 Table). The last-to-expire patent was a device patent on 39% (22/56) of products and 67% of (22/33) of drug-device combinations.

**Fig 2:**
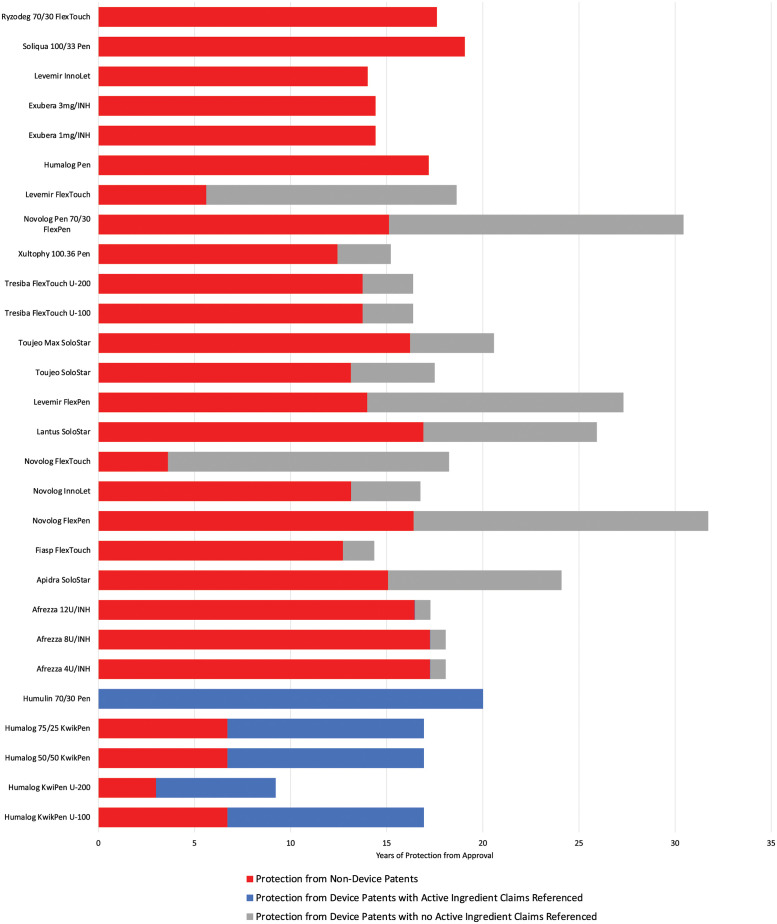
Role of device patents in extending protection on insulin drug-device combinations. This figure shows the patent protection on insulin products from non-device patents (red), such as patents on the active ingredients or methods of use, and the added protection afforded by patents on delivery devices that make mention of insulin (blue) versus those that make no mention of insulin (grey). Of the 33 drug-device combinations in the cohort, 5 were excluded from this figure since no patents were listed on these drug-device combinations in the Orange Book.

Of all patents on the 33 drug-device combinations in the cohort, 63% were on the delivery devices. The 22 last-to-expire device patents extended the duration of protection beyond non-device patents by a median of 5.2 years (IQR 2.6 to 10.2). Among the 22 last-to-expire device patents on these products, 17 (77%) made no mention of the active ingredient (insulin); these patents extended the duration of protection beyond other patents (device patents mentioning insulin or non-device patents) by a median of 4.3 years (IQR 2.6 to 13.0) (Fig 2). Of the 67 unique device patents in the cohort, 57 (85%) made no mention of insulin in their claims.

**Fig 3:**
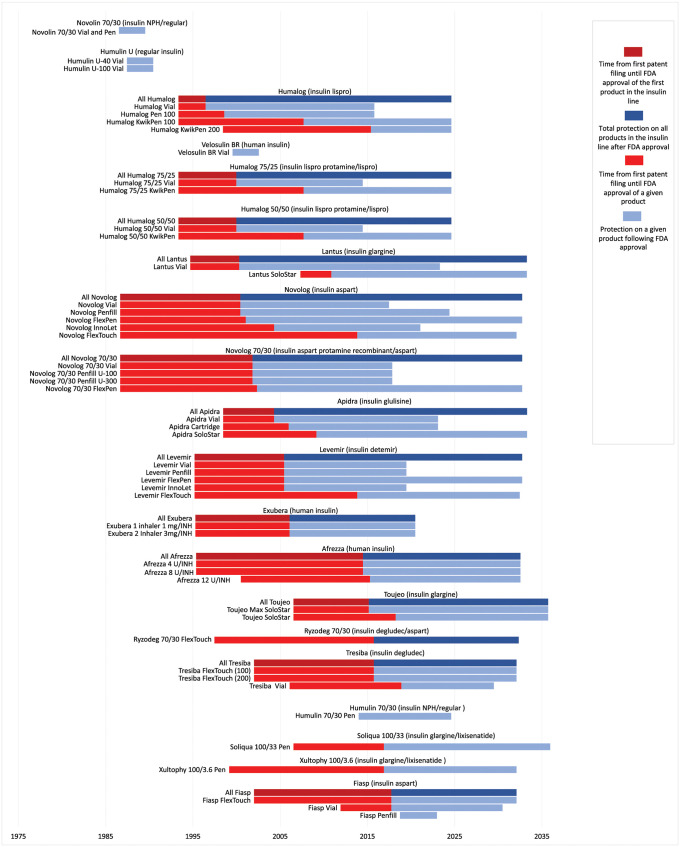
Brand-name originator insulins approved from 1986–2019. The figure shows how manufacturers extended protection on insulin products by releasing new versions with the same active ingredients. The light red bars show the time that elapsed from the first patent filing for an individual product until FDA approval of that product, and the light blue bars show the time from FDA approval of the product until expiration of the last-to-expire patent or regulatory exclusivity. The dark red bars show the time that elapsed from the first patent filing on all products approved under a given NDA, and the dark blue bars show the time from the first approval of a product under an NDA until expiration of the last-to-expire patent or regulatory exclusivity on any product approved under the NDA. Grey vertical lines represent decades in time. This analysis excludes products lacking patents or regulatory exclusivities at approval (Novolin R, Novolin L, Humulin BR, Novolin N, Humulin 50/50, the Humulin 70/30 vial, the Humalog 50/50 pen, and the Humalog 75/25 pen). In the case of Humalog, 5 products were approved under one NDA and 1 product under another NDA; for the purposes of this analysis, we treated these as a single product line when examining the total duration of protection.

Manufacturers frequently moved the same active ingredients to new products with different volumes, concentrations, and modes of delivery, all covered under the same NDA (Fig 3). When considering the median time of protection from the approval of the first brand-name drug in a given insulin line to the last-to-expire regulatory exclusivity or patent for all products in the insulin line, the median duration of protection was 17.6 years (IQR 3.0 to 24.6) (Fig 3). The insulin lines with the longest periods of expected protection from the first product approved to last-to-expire patent was Lantus (32.9 years), followed by Novolog (32.3 years) and Novolog 70/30 (30.9 years).

### Sensitivity analysis

Three follow-on insulin lines were approved during the study period, two on originators with active patents still listed and one on an originator after all patents had expired. Following successful challenges, Basaglar (glargine) received FDA approval 17.3 years before the last-to-expire patent or exclusivity on the originator Lantus. Admelog (lispro) received FDA approval 6.7 years before the last-to-expire patent or exclusivity on the originator Humalog. Myxredlin was distinct in that it received FDA approval after expiration of any patents or regulatory exclusivities on the originator.

After subtracting time lost to early entry of follow-ons for Lantus and Humalog, the median duration of protection on the 56 individual insulin products fell from 16.0 to 15.6 years (IQR 7.5 to 17.7). When considering protection for insulin lines stratified by NDA (sensitivity analysis), the median duration of protection after subtracting competition from follow-ons was 17.6 years (IQR 3.0 to 21.5 years), compared to 17.6 years of expected protection before subtracting time lost to early entry of follow-ons.

## Discussion

From 1986–2019, manufacturers of brand-name insulin products secured long periods of market protection and faced little direct competition from follow-on products. Protection on insulin was enhanced by patents obtained after FDA approval, which lengthened expected market exclusivity in 9 cases by a median of 6 years. In addition, two-thirds of drug-device combinations had last-to-expire patents that were on the delivery devices; these last-to-expire device patents extended protection by a median of 5.2 years. Overall, manufacturers secured a median of 16 years of protection on their insulin products through patents and exclusivities, surpassing the median of 14 years observed in other studies of top-selling small-molecule drugs [37]. Some of the most widely used insulins [38,39], such as glargine and degludec, were among those with the longest periods of market exclusivity.

This work documents the patenting and development practices that have extended periods of market exclusivity on brand-name insulin products over the last 35 years. The use of device patents has been a key factor driving the prolonged extension of these market protections, while products brought to market in the early years of the study period were rarely protected by device patents. This increased reliance on device patents is consistent with observations from markets for other drug-device combinations, including inhalers for asthma and chronic obstructive pulmonary disease [19,40] and GLP-1 receptor agonist injector pens for diabetes and obesity [41]. Innovations on delivery devices may benefit patients by making injectables easier to self-administer and reducing errors. Yet, these incremental innovations can also allow brand-name manufacturers to avoid direct competition and thereby keep prices high.

Even the limited follow-on competition observed in the insulin market has been driven by the big 3 brand-name manufacturers. The follow-on approved for Sanofi’s Lantus (glargine) was Lilly’s Basaglar (glargine), and the follow-on approved for Lilly’s Humalog (lispro) was Sanofi’s Admelog (lispro). Since the end of follow-up in our study, the FDA approved the first 2 interchangeable biologics, both for Lantus (glargine): Mylan’s Semglee (glargine) in July 2021 [42] and Lilly’s Rezvoglar (glargine) in December 2021 [43].

Approval of these competitors may eventually have substantial price lowering effects in the insulin market. However, to date, follow-on and biosimilar insulin uptake has been limited. Medicare Part D beneficiaries filled more prescriptions for brand-name Lantus in 2021 (the most recent year with publicly available data) than follow-on Basaglar [44]. Through the first quarter of 2023, the biosimilar Semglee represented fewer than 20% of glargine prescriptions in commercial markets and fewer than 10% in Medicare Part D [45,46]. Brand-name firms often maintain market share by negotiating rebates with pharmacy benefit managers (PBMs) in return for favorable formulary placement. Yet, patients still face high out of pocket since deductibles and co-insurance are typically based on pre-rebate list prices [47–51]. Expediting biosimilar availability may be an important step forward in lowering prices, but it may not suffice when only a small number of competitors have entered the market and PBMs have incentives to design formularies favoring originator biologics over biosimilars.

Manufacturers of several brand-name insulins recently announced that they would drop list prices in 2024, in part to avoid new penalties that will apply in Medicaid [8,52,53]. While such price reductions are a positive development, they apply only to a handful of insulin products. Price negotiation under the Inflation Reduction Act may also help lower brand-name insulin costs in Medicare, but the negotiated prices will not apply to commercial markets [54]. Timely robust generic and biosimilar competition—and payer reform to ensure patient access to these products—remain vital for lowering prescription drug prices in the US.

The role of device patents in delaying competition on insulin products has come under increased scrutiny in recent antitrust litigation. In a class-action lawsuit, a group of direct purchasers sued Sanofi for improperly listing a device patent on Lantus (glargine) in the Orange Book [20]. The case concerned a patent on the drive mechanism in the SoloStar pen that Sanofi added after approval. When Eli Lilly sought approval for the follow-on Basaglar in 2013, Sanofi sued for patent infringement. Sanofi eventually settled with Eli Lilly to allow for marketing of Basaglar at the end of 2016 and later reached settlements with Mylan and Merck in similar lawsuits. But the lengthy litigation that resulted from Sanofi’s patent on Lantus led to delays in competition for Lantus and substantial revenue for the company. In 2014 alone, when Basaglar approval was stalled due to litigation, Sanofi made several billion in US sales on Lantus [20]. In 2020, the US Court of Appeals for the First Circuit held that Sanofi’s patent on the drive mechanism of the SoloStar pen had been improperly listed in the Orange Book since it made no mention of insulin glargine, and the case has since been remanded for question of antitrust injury [20]. Our study identified 17 cases in which the last-to-expire patent on a drug-device combination was a device patent with claims that made no mention of insulin, and these patents extended protection by a median of more than 4 years. Regulatory uncertainty remains about which types of device patents manufacturers should list in the Orange Book [28]. But, as with earlier work on inhalers [21], our study underscores the prevalence of key device patents listed on products in the Orange Book with no mention of active ingredients in their claims. The Federal Trade Commission recently announced plans to scrutinize these types of patent listings [55].

The roots of limited competition in the insulin market go beyond the patenting practices that our study and others highlight [56]. The nebulous regulatory status of insulin prior to 2020—when it was treated as a small-molecule drug even though it possessed the properties of a biologic—meant that competitors could not pursue Abbreviated New Drug Applications for generic approvals or Biologics Licensing Applications for biosimilar approval [57,58]. Because approvals via the 505(b)(2) pathway are not automatically interchangeable with originators, the incentives for competitors to challenge patents were diminished. Though manufacturers of originator insulins earned sizeable revenue, the risks of costly litigation and concerns of limited uptake may have deterred competitors. A systematic examination of how brand-name firms used litigation to stall generic and biosimilar entry goes beyond the scope of this paper, but the extensive patenting on insulin products has clearly contributed to delays in competition.

The move to regulate insulin as a biologic comes with its own obstacles. One is that manufacturers now need not list all key patents with the FDA (as they were required to do when insulin was regulated as a small-molecule drug in the Orange Book) but instead must only list litigated patents in an alternative FDA-published compendium known as the Purple Book. This may make it difficult for biosimilar manufacturers to challenge new insulin products in the coming years (or even older products given the churn of newly added patents and the unknown legal costs due to the opacity of patent protections). Requiring biologic manufacturers to list all key patents with the FDA, as some have proposed, would help address this problem [59].

A second barrier for biosimilars is that the threshold for issuing new drug-related patents at the USPTO may be too low—particularly for the device patents that often predominate on drug-device combinations. Other patent agencies, like the European Patent Office, tend to apply more exacting standards to these patents [60]. The USPTO could increase the quality of issued patents by incorporating decisions of these comparable international patent offices (for example, by flagging patents that have been rejected or withdrawn in those offices) [16] and by creating a special unit within the patenting office dedicated to examining patents on drug-device combinations [61]. Further collaboration between the FDA and USPTO, now a priority for both agencies [27], could also help limit abuses of the patent system.

Our analysis has several important limitations. First, the analysis may understate the protection afforded by patents and regulatory exclusivities since brand-name firms can continue to add patents to existing products in the future. On the other hand, future competitors may enter the market before patent expiration, as was seen with Lantus and Humalog, which could lead us to overestimate expected patent protection. Unless the FDA begins to require more comprehensive patent listings for biologics in the Purple Book, the window that we have into patent protection for insulin products moving forward will be tightly circumscribed. Second, our analysis was descriptive; further empirical analyses are needed to probe the multitude of factors associated with delayed entry of competitor insulin products. Third, we only examined patenting practices on insulin products rather than other non-insulin therapies. Fourth, our analysis focused only on the US patent system. Recent work has identified many device patents on drug-device combination products in Canada, the European Union, Japan, Australia, and several other high-income countries [62]. Prices for insulin around the world, including in developing countries, remain high [5,63–65]; the World Health Organization has identified improved global access to insulin and other diabetes therapies as a key public health priority in the coming decades [66]. Future work should seek to understand how the international patent system may hinder access to lower-cost biosimilar insulins. Finally, while our analysis emphasized how patenting practices on drug-device combinations may delay competition, we did not assess other impacts; single-use drug-device combinations, for example, can have significant environmental impact by contributing to waste.

Insulin manufacturers have employed several strategies to extend periods of market exclusivity on brand-name insulin products, particularly filing patents after FDA approval and obtaining a large number of patents on delivery devices. In conjunction with recently passed legislation to help rein in prices on insulin, Congress, the FDA, and the USPTO should continue to work on reforming the patent and regulatory system to prevent such thickets from continuing to interfere with a healthy pipeline of cheaper—and even interchangeable—insulin products for the benefit of patients.

## Supporting information

S1 Methods Many insulin products approved during the study period were drug-device combinations. (PDF)

S1 Table Animal-derived insulin products approved in 1986 or later. (PDF)

S2 Table Biosynthetic insulin products approved before 1986. (PDF)

S3 Table Originator insulin products approved from 1986–2019. (PDF)

S4 Table Follow-on insulins products approved from 1986–2019. (PDF)

S5 Table Exclusivities obtained by manufacturers on insulin products at FDA approval. (PDF)

S6 Table Sensitivity analysis for patents listed on brand-name originator insulins. (PDF)

S7 Table Products qualifying for pediatric exclusivities. (PDF)

S8 Table Exclusivities obtained by manufacturers on insulin products after approval. (PDF)

S9 Table Duration of market protection on insulin products. (PDF)

## Abbreviations

FDA: Food and Drug Administration
GLP-1: glucagon-like peptide 1
NBER: National Bureau for Economic Research
NDA: New Drug Application
PBM: pharmacy benefit manager
SGLT2: sodium-glucose cotransporter 2
USPTO: US Patent and Trademark Office
