# Supplementary material for: Patents and regulatory exclusivities on FDA-approved insulin products: A longitudinal database study, 1986–2019
Source: PLoS Med. 2023 Nov 16;20(11):e1004309. doi: 10.1371/journal.pmed.1004309 (PMC10653475; doi:10.1371/journal.pmed.1004309)
Supplement: S1 Methods — (PDF) [file pmed.1004309.s001.pdf]

S1 Methods

Many insulin products approved during the study period were drug-device combinations. The FDA regulates drug-device combinations as either “drug-led combinations” or “device-led combinations” depending on which constituent provides the primary mode of action [1]. For example, an albuterol inhaler is regulated as a drug-led combination since the drug is what acts on receptors in the lungs, while a drug-eluting stent is regulated as a device-led combination since the stent is what maintains patency of an artery. The FDA has treated insulin products sold in pens and inhalers as drug-led combinations; all such combinations are included in our analysis. By contrast, our analysis excludes devices that are sold separately from insulins, as these are approved through the FDA’s medical device pathway and are not part of the Orange Book [2].

The study included biosynthetic insulin products that were approved by the FDA from 1986 to 2019. However, two products that were approved in late 2019 (Humalog KwikPen 200 and Humalog Tempo Pen 1000) were excluded, because patents and regulatory exclusivities on these products did not make it into the 2020 Orange Book (and insulin products no longer appeared in the 2021 Orange Book or later versions since their regulatory status changed).

We considered individual products sold under the same New Drug Application (NDA)—for example, a vial and a pen—as separate products for the primary analysis. We also performed an analysis in which we analyzed the duration of protection from patents and regulatory exclusivities on all products in a given insulin line (i.e., under the same NDA). When performing this analysis, there was one case (Humalog) in which 5 products had been approved under one NDA and one product under another NDA; here, we analyzed all 6 products together when analyzing expected market protection for Humalog.

Three patents in the cohort were on delivery devices but were listed in the Orange Book on products that were not drug-device combinations. These patents were excluded from our analysis when examining the number of patents on these products and their periods of patent protection.

References

1. Principles of Premarket Pathways for Combination Products - Guidance for Industry and FDA Staff. Available online at: https://www.fda.gov/regulatory-information/search-fda-guidance-documents/principles-premarket-pathways-combination-products. Accessed September 27, 2023.
2. Warshaw H. A Look Inside the FDA's Review Processes for Medical Devices. *News & Publications* blog. Aug 30, 2021. Available online at: https://www.diabeteseducator.org/news/perspectives/aade-blog-details/adces-perspectives-on-diabetes-care/2021/08/30/fda-review-processes-for-medical-devices. Accessed September 27, 2023.
