# Supplementary material for: Patents and regulatory exclusivities on FDA-approved insulin products: A longitudinal database study, 1986–2019
Source: PLoS Med. 2023 Nov 16;20(11):e1004309. doi: 10.1371/journal.pmed.1004309 (PMC10653475; doi:10.1371/journal.pmed.1004309)
Supplement: S1 Table — (PDF) [file pmed.1004309.s002.pdf]

S1 Table: Animal-derived insulin products approved in 1986 or later

Brand	Insulin	NDA	Approval
Velosulin BR	Human insulin	N019450	05/30/1986
Novolin 70/30	Human insulin	N019441	07/11/1986
Mixtard 70/30	Human insulin	N019585	03/11/1988
Insulatard NPH	Human insulin	N019449	05/30/1986

NDA: New Drug Application
