# Supplementary material for: Patents and regulatory exclusivities on FDA-approved insulin products: A longitudinal database study, 1986–2019
Source: PLoS Med. 2023 Nov 16;20(11):e1004309. doi: 10.1371/journal.pmed.1004309 (PMC10653475; doi:10.1371/journal.pmed.1004309)
Supplement: S2 Table — (PDF) [file pmed.1004309.s003.pdf]

S2 Table: Biosynthetic insulins approved before 1986

Brand	Insulin	NDA	Approval
Humulin R	Regular insulin	N018780	10/28/1982
Humulin L	Regular insulin	N019377	09/30/1985
Humulin N	NPH	N018781	10/28/1982

NDA: New Drug Application
