# Supplementary material for: Patents and regulatory exclusivities on FDA-approved insulin products: A longitudinal database study, 1986–2019
Source: PLoS Med. 2023 Nov 16;20(11):e1004309. doi: 10.1371/journal.pmed.1004309 (PMC10653475; doi:10.1371/journal.pmed.1004309)
Supplement: S3 Table — (PDF) [file pmed.1004309.s004.pdf]

S3 Table: Originator insulin products approved from 1986-2019

Brand	Insulin	NDA	Number	Concentration	Product	Drug-device combination	Approval
Rapid-acting insulins							
**Apidra**	glulisine	N021629	001	U-100	Vial	No	04/16/2004
			002	U-100	Cartridge	No	12/20/2005
			003	U-100	SoloStar	Yes	02/24/2009
**Fiasp**	aspart	N208751	001	U-100	Vial	No	09/29/2017
			002	U-100	FlexTouch	Yes	09/29/2017
			003	U-100	PenFill	No	09/24/2018
**Humalog**[a]	lispro	N020563	001	U-100	Vial/ Cartridge	No	06/14/1996
			002	U-100	Pen	Yes	08/06/1998
			003	U-100	KwikPen[b]	Yes	09/06/2007
		N205747	001	U-200	KwikPen	Yes	05/26/2015
**Novolog**	aspart	N020986	001	U-100	Vial	No	06/07/2000
			002	U-100	PenFill	No	06/07/2000
			003	U-100	FlexPen	Yes	01/19/2001
			004	U-100	InnoLet	Yes	04/23/2004
			005	U-100	FlexTouch	Yes	10/31/2013
**Afrezza**	human	N022472	001	--	DPI	Yes	06/27/2014
			002	--	DPI	Yes	06/27/2014
			003	--	DPI	Yes	04/17/2015
**Exubera**	human	N021868	001	--	DPI	Yes	01/27/2006
			002	--	DPI	Yes	01/27/2006
**Velosulin BR**	human	N021028	001	U-100	Vial	No	07/19/1999
Short-acting insulins							
**Novolin R**[c]	regular	N019938	001	U-100	Multiple[d]	Yes	06/25/1991
**Novolin L**[c]		N019965	001	U-100	Vial	No	06/25/1991
**Humulin BR**	regular	N019529	001	U-100	Vial	No	04/28/1986
**Humulin U**		N019571	001	U-40	Vial	No	06/10/1987
		N019571	002	U-100	Vial	No	06/10/1987
Intermediate-acting insulins							
**Novolin N**[c]	NPH	N019959	001	U-100	Multiple[e]	Yes	07/01/1991
Long-acting insulins							
**Lantus**[f]	glargine	N021081	001	U-100	Vial	No	04/20/2000
			002	U-100	SoloStar	Yes	04/27/2007
**Levemir**	detemir	N021536	001	U-100	Vial	No	06/16/2005
			002	U-100	FlexPen	Yes	06/16/2005
			003	U-100	InnoLet	Yes	06/16/2005
			004	U-100	PenFill	No	06/16/2005
			005	U-100	FlexTouch	Yes	10/31/2013
**Toujeo**	glargine	N206538	001	U-300	SoloStar	Yes	02/25/2015
			002	U-300	Max SoloStar	Yes	03/26/2018
**Tresiba**	degludec	N203314	001	U-100	FlexTouch	Yes	09/25/2015
			002	U-200	FlexTouch	Yes	09/25/2015
			003	U-100	Vial	No	11/21/2018
Long-acting insulin and incretin mimetic							

**Xultophy 100/3.6**	glargine/ lixisenatide	N208583	001	U-100	Pen	Yes	11/21/2016
**Soliqua 100/33**	glargine/ lixisenatide	N208673	001	U-100	Pen	Yes	11/21/2016
**Intermediate and rapid-acting insulin mix**							
**Humalog 50/50**	lispro protamine/ lispro	N021018	001	--	Vial/ Cartridge[g]	No	12/22/1999
			002	--	KwikPen[b]	Yes	09/06/2007
			003	--	Pen	Yes	12/22/1999
**Humalog 75/25**	lispro protamine/ lispro	N021017	001	--	Vial/ Cartridge[g]	No	12/22/1999
			002	--	KwikPen	Yes	09/06/2007
			003	--	Pen	Yes	12/22/1999
**Novolog 70/30**	apart protamine/ aspart	N021172	001	--	Vial	No	11/01/2001
			002	--	PenFill	No	11/01/2001
			003	--	PenFill	No	11/01/2001
			004	--	FlexPen	Yes	05/03/2002
**Ryzodeg 70/30**	degludec/ aspart	N203313	001	--	FlexTouch	Yes	09/25/2015
**Intermediate and short-acting insulins**							
**Humulin 50/50**	NPH/ regular	N020100	001	--	Vial	No	04/29/1992
**Humulin 70/30**[a]	NPH/ regular	N019717	001	--	Vial	No	04/25/1989
			002	--	Pen	Yes	08/06/1998
**Novolin 70/30**[c]	NPH/ regular	N019991	001	--	Multiple[h]	Yes	06/25/1991

NDA: New drug application, DPI: Dry powder inhaler

a. Eli Lilly released vials of Humalog with different volumes (and different National Drug Codes [NDCs]) and cartridges all at the same U-100 strength. They did not list these products separately in the Orange Book but instead grouped them under NDA N020563 (product 001). They did the same for vials of Humulin 70/30 Mix (grouping them under N019717 [product 001]).

b. The KwikPen Junior is included under Eli Lilly's Humalog U-100 Kwikpen listing (NDA N020563 [product 003]) and Humalog 50/50 Kwikpen listing (NDA N021018 [product 002]).

c. Novolin R (approved 1983), Novolin L (1983), Novolin N (1985), and Novolin 70/30 (1986) were initially approved as semisynthetic versions (with different NDAs). The versions listed here are all biosynthetic versions.

d. Novo Nordisk released Novolin R products with National Drug Code (NDCs) that included the vial, Penfill, InnoLet, and FlexPen. However, these products are not listed separately in the Orange Book. Only one product is included under NDA N019938 (product 001).

e. Novo Nordisk released Novolin N products with NDCs that included the vial, Penfill, InnoLet, and FlexPen. However, these products are not listed separately in the Orange Book. Only one product is included under NDA N019959 (product 001).

f. Sanofi grouped its vial of Lantus and cartridge under NDA N021081 (product 001).

g. Eli Lilly released Humalog 50/50 Mix and Humalog 75/25 Mix as both a vial and cartridge (with different NDCs) at the same strength. They did not list these products separately in the Orange Book but instead grouped them under NDA N021018 (product 001).

h. Novo Nordisk released Novolin 70/30 products with NDCs that include the vial, Penfill, InnoLet, and FlexPen. However, these products are not listed separately in the Orange Book. Only one product is included under NDA N019959 (product 001).
