# Supplementary material for: Patents and regulatory exclusivities on FDA-approved insulin products: A longitudinal database study, 1986–2019
Source: PLoS Med. 2023 Nov 16;20(11):e1004309. doi: 10.1371/journal.pmed.1004309 (PMC10653475; doi:10.1371/journal.pmed.1004309)
Supplement: S4 Table — (PDF) [file pmed.1004309.s005.pdf]

S4 Table: Follow-on insulins products approved from 1986-2019

**Brand**	**Insulin**	**NDA**	**Number**	**Concentra tion**	**Product**	**Drug-device combin ation**	**Approval**
**Rapid-acting insulins**							
**Admelog**	lispro	N209196	001	U-100	Vial	No	12/19/2018
	lispro	N209196	002	U-100	SoloStar	Yes	12/11/2017
	lispro	N209196	002	U-300	Vial	No	12/11/2017
**Myxredlin**	human	N208157	001	--	Infusion bag	No	06/20/2019
**Long-acting insulins**							
**Basaglar**	glargine	N205692	001	U-100	KwikPen	Yes	12/16/2015
