# Supplementary material for: Patents and regulatory exclusivities on FDA-approved insulin products: A longitudinal database study, 1986–2019
Source: PLoS Med. 2023 Nov 16;20(11):e1004309. doi: 10.1371/journal.pmed.1004309 (PMC10653475; doi:10.1371/journal.pmed.1004309)
Supplement: S5 Table — (PDF) [file pmed.1004309.s006.pdf]

S5 Table: Exclusivities obtained by manufacturers on insulin products at FDA approval

Category of exclusivity at approval	Number of products	Products granted exclusivity
NCE: New chemical entity (5 years)<br>Granted for newly discovered chemical or molecular entities.	11	Apidra Vial (glulisine)
		Humalog Vial (lispro)
		Humalog Pen (lispro)
		Novolog Vial (aspart)
		Lantus Vial (glargine)
		Levemir Vial (detemir)
		Tresiba Pen FlexTouch 100 (degludec)
		Tresiba Pen FlexTouch 200 (degludec)
		Xultophy Pen (glargine/ lixisenatide)
		Soliqua Pen (glargine/ lixisenatide)
		Ryzodeg FlexTouch (degludec/aspart)
NP: New product (3 years)<br>Granted for new products based on previously approved products.	10	Fiasp Vial (aspart)
		Fiasp FlexTouch (aspart)
		Afrezza 4U/INH (human)
		Afrezza 8U/INH (human)
		Afrezza 12U/INH (human)
		Exubera 1mg/INH (human)
		Exubera 3mg/INH (human)
		Humulin U Vial 40 U/mL (regular)
		Humulin U Vial 100 U/mL (regular)
		Toujeo SoloStar (glargine)
NC: New combination (3 years)<br>Granted for new combinations of existing products.	6	Xultophy Pen (glargine/ lixisenatide)
		Soliqua Pen (glargine/ lixisenatide)
		Humalog 50/50 Vial (lispro protamine/lispro)
		Humalog 75/25 Vial (lispro protamine/lispro)
		Novolog 70/30 Vial (protamine/aspart)
		Novolin 70/30 Vial and Pen (NPH/regular)
NPP: New patient population (3 years)<br>Granted for new indications for patient populations.	1	Apidra SoloStar (glulisine)
