# Supplementary material for: Patents and regulatory exclusivities on FDA-approved insulin products: A longitudinal database study, 1986–2019
Source: PLoS Med. 2023 Nov 16;20(11):e1004309. doi: 10.1371/journal.pmed.1004309 (PMC10653475; doi:10.1371/journal.pmed.1004309)
Supplement: S6 Table — (PDF) [file pmed.1004309.s007.pdf]

S6 Table: Sensitivity analysis for pre-approval patents listed on brand-name originator insulins[a]

Period of approval	Number of products approved per period	Median (IQR) patents listed per product	Median (IQR) device patents listed per product
1986-1990	5	0 (0-0)	0 (0-0)
1991-1995	4	0 (0-0)	0 (0-0)
1996-2000	11	2 (0-4)	0 (0-0)
2001-2005	12	5 (4-7)	0 (0-0)
2006-2010	7	5 (4-9)	1 (1-3)
2011-2015	10	17 (15-32)	15 (12-15)
2016-2019[b]	7	19 (1-24)	18 (0-19)

IQR: Interquartile range

a. The primary analysis examined trends in the number of pre-approval patents listed on brand-name originators approved during different tertiles of the study period. Because each period was more than a decade in duration, this choice could have masked certain trends within a given time period. We therefore performed a sensitivity analysis analyzing pre-approval patents during 5-year increments and observed similar findings.

b. This period was only 4 years since the study period included only product approved through the end of 2019.
