# Supplementary material for: Patents and regulatory exclusivities on FDA-approved insulin products: A longitudinal database study, 1986–2019
Source: PLoS Med. 2023 Nov 16;20(11):e1004309. doi: 10.1371/journal.pmed.1004309 (PMC10653475; doi:10.1371/journal.pmed.1004309)
Supplement: S7 Table — (PDF) [file pmed.1004309.s008.pdf]

S7 Table: Products qualifying for pediatric exclusivities

Category of exclusivity after approval	Number of products	Products granted exclusivity
PED: Pediatric exclusivities are granted for products that have FDA approval on treatments that can produce health benefits for pediatric populations (6 months)	10	Novolog Vial (aspart)
		Novolog Innolet (aspart)
		Novolog Flextouch (aspart)
		Lantus Vial (glargine)
		Toujeo SoloStar (glargine)
		Xultophy Pen (glargine/ lixisenatide)
		Novolog 70/30 Vial (aspart protamine/aspart)
		Novolog 70/30 Penfill 002 (aspart protamine/aspart)
		Novolog 70/30 Penfill 003 (aspart protamine/aspart)
		Novolog 70/30 Flexpen (aspart protamine/aspart)
