# Supplementary material for: Patents and regulatory exclusivities on FDA-approved insulin products: A longitudinal database study, 1986–2019
Source: PLoS Med. 2023 Nov 16;20(11):e1004309. doi: 10.1371/journal.pmed.1004309 (PMC10653475; doi:10.1371/journal.pmed.1004309)
Supplement: S8 Table — (PDF) [file pmed.1004309.s009.pdf]

S8 Table: Exclusivities obtained by manufacturers on insulin products after approval

Category of exclusivity after approval	Number of products	Products granted exclusivity
NC: New Combination (3 years) Granted for new combinations of existing products.	2	Tresiba Pen FlexTouch 100
		Tresiba Pen FlexTouch 200
NPP: New patient population (3 years) Granted for new indications for patient populations.	8	Apidra Vial 300U/3mL (glulisine)
		Apidra Vial 1,000U/3mL (glulisine)
		Fiasp Vial (aspart)
		Fiasp Penfill (aspart)
		Fiasp FlexTouch (aspart)
		Toujeo SoloStar (glargine)
		Toujeo Max SoloStar (degludec)
		Ryzodeg FlexTouch (degludec/aspart)
NR: New route (3 years) Granted for new routes of administration.	3	Humalog Vial (lispro)
		Humalog Pen (lispro)
		Novolog Vial (aspart)
NP: New product (3 years) Granted for new products based on previously approved products.	1	Velosulin BR Vial (human)
D-56: Addition of post-prandial dosing (3 years)	2	Humalog Vial (lispro)
		Humalog Pen (lispro)
D-80: Change of dosing schedule for Lantus from once daily to at bedtime to flexible daily dosing (3 years)	1	Lantus Vial (glargine)
D-86: For Use in Select External Insulin Pumps (3 years)	2	Humalog Vial (lispro)
		Humalog Pen (lispro)
D-112: Provides for pediatric pump use (3 years)	1	Novolog Vial
I-489: For use in pediatrics patients with Type 1 diabetes (3 years)	1	Levemir Vial (detemir)
M-44: Clinical information added to the pediatric use subsection of precautions regarding the use of Novolog in adolescents with Type 1 diabetes age 6 to 18 (3 years)	1	Novolog Vial (aspart)
M-48: Changes to labeling describing the results of a study of the use of Novolog mix 70/30 with oral antidiabetic agents in patients with Type 2 diabetes (3 years)	1	Novolog 70/30 Vial (aspart protamine/aspart)
M-115: Revisions based on the PI based on results from study NN2211-1842, entitled the effect of insulin detemir in combination with liraglutide and metformin compared to liraglutide and metformin in subjects with Type 2 diabetes (3 years)	1	Levemir Vial (detemir)
M-117: Addition of results of pediatric trial to label (3 years)	1	Levemir Vial (detemir)
M-242: Information Added Regarding the Efficacy and Safety of Insulin	1	Xultophy Pen (glargine/ lixisenatide)

Degludec/Liraglutide vs Insulin Glargine in Patients with Type 2 Diabetes Mellitus Inadequately Controlled on SGLT2 inhibitors with or without Oral Antidiabetic Therapies (3 years)		
M-247: Revisions to the labeling regarding continuous subcutaneous insulin infusion as a condition of use for insulin Aspart (3 years)	1	Fiasp Vial (aspart)

Orange book codes that start with “D” refer to dosing-related exclusivities; codes that start with “I” refer to indication-related exclusivities; codes that start with “M” refer to miscellaneous exclusivities.
*Multiple pediatric extensions can be placed on a singular product
