# Supplementary material for: Patents and regulatory exclusivities on FDA-approved insulin products: A longitudinal database study, 1986–2019
Source: PLoS Med. 2023 Nov 16;20(11):e1004309. doi: 10.1371/journal.pmed.1004309 (PMC10653475; doi:10.1371/journal.pmed.1004309)
Supplement: S9 Table — (PDF) [file pmed.1004309.s010.pdf]

S9 Table: Duration of market protection on insulin products

**Class**	**Products in class**	**Median (IQR) protection from FDA approval to last-to-expire patent or regulatory exclusivity, years**
Rapid-acting	21	17.1 (14.4-18.3)
Short-acting	5	3.0 (3.0-3.0)
Intermediate-acting	1	0.0 (0.0-0.0)
Long-acting	12	16.9 (14.0-21.2)
Long-acting and incretin mimetic	2	17.1 (16.2-18.1)
Intermediate and rapid-acting mix	11	15.6 (14.5-16.8)
Intermediate and short-acting mix	4	3.0 (1.5-11.5)
**Total**	**56**	**16.0 (10.3-18.1)**

IQR: Interquartile range, FDA: Food and Drug Administration
